# Supplementary material for: The association of denture wearing with reduced lung function and increased airflow limitation in 58–72 year old men
Source: PLoS One. 2023 May 18;18(5):e0285117. doi: 10.1371/journal.pone.0285117 (PMC10194954; doi:10.1371/journal.pone.0285117)
Supplement: Attachment — Submitted filename: Response to reviewers.docx [file pone.0285117.s003.docx]

**Response to reviewers**

We are very grateful to the academic editor and the referees for carrying out a thorough review of our submitted article “The association of denture wearing with reduced lung function and increased airflow limitation in 58-72 year old men”. We have attempted to address all suggestions and revised the manuscript accordingly. We believe that the revised manuscript is a significant improvement on the original and are very happy to acknowledge the reviewers’ role in this. We hope that the revised version will now be suitable for publication in PLOS One.

**Response to the Academic Editor**

As requested we have made changes to the financial disclosures.

The revised financial disclosure statement will now read: This work was funded by the Northern Ireland Research and Development fund as grants RRG 1.2 and RRG 5.22 (PI: Linden). The funding body has since been renamed as the HSC Research and Development Division, Public Health Agency, Northern Ireland (Web address: research.hscni.net). The funders had no role in study design, data collection and analysis, decision to publish, or preparation of the manuscript.

We have revised the title page to meet the requirements of PLOS ONE.

We have revised the body of the text and the tables to meet the requirements of PLOS ONE.

We acknowledge our error in relation to failing to forward a minimum dataset. We have added a supporting information section and provided an Excel file “S1.Minimum dataset”. The data availability statement should now read: All relevant data are within the paper and its Supporting information files.

We have added a section detailing the author contributions.

In the revised marked-up version of the manuscript text changes are outlined in green.

**Specific responses to the reviewers**

In general we thank the referees for the very positive comments that they made about our manuscript.

**Reviewer #1**: We agree with the broad thrust of the referee’s comments regarding the limitations of the study. Nevertheless, when we identified the association between denture wearing and poorer lung function we believed it was important to report it, despite the limitations. We accept that with knowledge of hindsight it would have been beneficial to collect further data on denture wearing and cleaning habits. Such denture-related data would have facilitated further investigation of the influence of these variables, however, since the comparison was between denture wearers and never denture wearers we would contend that the conclusions we have drawn are supported by our analysis of the available data. We have tried to be as up-front as possible about the limitations. In general these relate to decisions made many years ago and driven by the focus of the PRIME Study where there was limited time available to complete the investigations (blood sampling, ECG, lung function, physical examination, OPT radiograph, on-site questionnaire completion) of each participant.

The men recruited to the Belfast PRIME study were 50-60 years old at their initial examination and in the general description of the study it suggests that they were examined in 1991-1992. However, it was the case that a number of the examinations did not take place until 1993 and 1994. When the men were re-screened between 2001-2004 in some cases it had been less than 10 years since their initial examination and in others it was more than 10 years. As a result a small proportion of the men (5.6%) fell outside the 60-70 year-old age bracket. We hope this clarifies the query raised.

We apologise for the oversight regarding 2 citations in the introduction. We have now inserted references 14,15.

We have modified Table 1 as requested.

**Reviewer #2**: We appreciate the very positive comments on our manuscript.

**Reviewer #3**: We have now made the data available as outlined above.

We thank the referee for the marked-up version of the paper and have made the minor corrections. We can confirm that there were no implant-supported dentures.
